# Expression of TRPV1 Channels after Nerve Injury Provides an Essential Delivery Tool for Neuropathic Pain Attenuation

**DOI:** 10.1371/journal.pone.0044023

**Published:** 2012-09-04

**Authors:** Hossain Md. Zakir, Rahman Md. Mostafeezur, Akiko Suzuki, Suzuro Hitomi, Ikuko Suzuki, Takeyasu Maeda, Kenji Seo, Yoshiaki Yamada, Kensuke Yamamura, Shaya Lev, Alexander M. Binshtok, Koichi Iwata, Junichi Kitagawa

**Affiliations:** 1 Division of Oral Physiology, Department of Oral Biological Science, Niigata University Graduate School of Medical and Dental Sciences, Niigata, Japan; 2 Division of Oral Anatomy, Department of Oral Biological Science, Niigata University Graduate School of Medical and Dental Sciences, Niigata, Japan; 3 Department of Physiology, Nihon University School of Dentistry, Tokyo, Japan; 4 Division of Dental Anesthesiology, Department of Oral Biological Science, Niigata University Graduate School of Medical and Dental Sciences, Niigata, Japan; 5 Department of Medical Neurobiology, Institute for Medical Research Israel Canada and Center for Research on Pain, The Hebrew University Medical School, Jerusalem, Israel; Universidad Federal de Santa Catarina, Brazil

## Abstract

Increased expression of the transient receptor potential vanilloid 1 (TRPV1) channels, following nerve injury, may facilitate the entry of QX-314 into nociceptive neurons in order to achieve effective and selective pain relief. In this study we hypothesized that the level of QX-314/capsaicin (QX-CAP) - induced blockade of nocifensive behavior could be used as an indirect in-vivo measurement of functional expression of TRPV1 channels. We used the QX-CAP combination to monitor the functional expression of TRPV1 in regenerated neurons after inferior alveolar nerve (IAN) transection in rats. We evaluated the effect of this combination on pain threshold at different time points after IAN transection by analyzing the escape thresholds to mechanical stimulation of lateral mental skin. At 2 weeks after IAN transection, there was no QX-CAP mediated block of mechanical hyperalgesia, implying that there was no functional expression of TRPV1 channels. These results were confirmed immunohistochemically by staining of regenerated trigeminal ganglion (TG) neurons. This suggests that TRPV1 channel expression is an essential necessity for the QX-CAP mediated blockade. Furthermore, we show that 3 and 4 weeks after IAN transection, application of QX-CAP produced a gradual increase in escape threshold, which paralleled the increased levels of TRPV1 channels that were detected in regenerated TG neurons. Immunohistochemical analysis also revealed that non-myelinated neurons regenerated slowly compared to myelinated neurons following IAN transection. We also show that TRPV1 expression shifted towards myelinated neurons. Our findings suggest that nerve injury modulates the TRPV1 expression pattern in regenerated neurons and that the effectiveness of QX-CAP induced blockade depends on the availability of functional TRPV1 receptors in regenerated neurons. The results of this study also suggest that the QX-CAP based approach can be used as a new behavioral tool to detect dynamic changes in TRPV1 expression, in various pathological conditions.

## Introduction

Neuropathic pain (NP), which may arise as a result of injury, inflammation, or disease of the peripheral or central nervous systems, is characterized by spontaneous pain (i.e. ongoing, paroxysmal) and evoked sensitization in the form of hyperalgesia or allodynia. The TRPV1 channel, which is classically associated with transduction of painful stimuli such as hot temperature, low pH and application of vanilloid substances [1], [2], [3], [4] has been shown to change its expression profile under neuro-pathological conditions. Such changes have been implicated in neuropathic pain, by underlying changes in neuronal excitability [5], [6], [7], [8], [9], [10]. Several reports have described changes in TRPV1 expression levels in neuropathic pain models. Decrease of TRPV1 levels in injured and increased expression of TRPV1 in uninjured or spared neurons, was reported to occur after nerve ligation/transection [5], [6], [7], [8], [9], [10], however, the dynamics of functional TRPV1 expression during regeneration of transected nerves in this respect is still elusive. This information is highly important when exploring therapeutically relevant avenues in which TRPV1 may play an essential role. In naïve animals, TRPV1 is exclusively expressed in peripheral C- and Aδ- fibers [1]. Contrary to its role as a transducer in pain fibers, TRPV1 has been shown to serve also as a carrier for selective blockers of excitability. Blocking pain fibers specifically can be achieved by exploiting the selective TRPV1 expression in these fibers and the ability to use TRPV1 as a carrier of neuronal excitability blockers such as the non-permeable sodium channel blocker N-(2,6-dimethylphenylcarbamoylmethyl) triethylammonium bromide (QX-314). QX-314 is a permanently positively charged sodium channel blocker, which is unable to readily cross the cell membrane in a passive manner [11], [12], [13], [14]. However, when opening the TRPV1 channel by capsaicin, QX-314 can enter and thereby block nociceptive sodium channels from the inside of the cell, producing a long-lasting, pain-specific local anesthesia, devoid motor or tactile deficits [15], [16], [17], [18]. Based on the fact that TRPV1 plays a major role in this strategy, we explored whether the combination of QX-314 together with capsaicin (QX-CAP) could be used, not only to understand the dynamic functional expression of TRPV1 during regeneration of injured nerves, but also to block nerve injury mediated hyperalgesia. We further examined where (i.e. cell types) TRPV1 is expressed following IAN transection and nerve regeneration. This information is essential for better understanding mechanisms of pain, and thereby allowing development of novel strategies to manage pain. In this study we used the combination of QX-314 and capsaicin that was developed for selective blocking of pain [15], [16], [17] to understand the functional expression of TRPV1 in conjunction with profiling TRPV1 expression by immunohistochemistry. We show that starting 3 weeks after nerve transection, the QX-CAP combination reduces the hypersensitivity in the area of nerve regeneration and that this is dependent upon the amount of nerve regeneration in the injured area and the level of TRPV1 expression in these nerves. We further show a shift in the expression of TRPV1 from non-myelinated regenerated nerves to myelinated regenerated nerves and compare this data between those animals which underwent hyperalgesia and those which did not, as result of induced nerve transection (as a model for nerve injury induced neuropathic pain) hinting to the fact that other fibers besides nociceptive fibers participate in inducing pain sensation. Finally we propose using the behavioral testing as a tool to qualitatively relay relative TRPV1 expression levels after nerve injury is initiated.

For the first time we can give an accurate account not only of changes in TRPV1 expression (both in time and cell type) but cross correlate this to behavioral testing and blockade of painful sensation. This novel approach for targeted painful stimuli in a neuropathic pain model can serve as a platform to be developed into clinically relevant strategies for pain management.

## Methods

The experiments were carried out in accordance with the guidelines of National Institute of Health Guide for the Care and Use of Laboratory (NIH Publication no. 80–23) revised 1996 and the International Association for the Study of Pain in conscious Animals, and were approved by the intramural Animal Care and Veterinary Science Committee of Niigata University [19]. Surgery was performed under sodium pentobarbital anesthesia, and all efforts were made to minimize suffering.

A total of 120 male rats (Sprague-Dawley), weighing 150–200 grams at the start of the experiment, were used. The rats were exposed to a light dark cycle of 12 hours. Food and water were available *ad libitum*.

### IAN Transection and Sham Operation

Rats were anesthetized with sodium pentobarbital (50 mg/kg, administered intraperitoneally (IP)), which was proceeded with left IAN transection. In this procedure, the rats were placed on a warm mat (to control for normal bodily temperature) and a small incision was made in the facial skin over the masseter muscle. The muscle was dissected to expose the surface of the alveolar bone. The bone covering IAN was removed using a dental drill. The exposed IAN was lifted, transected, and then placed back in the mandibular canal without any discernible gap between the cut ends [20], [21].

Rats with a similar facial skin dissection but without IAN exposure and transection were categorized as the sham-operated group (QX-CAP administration: *n* = 15, CAP administration: *n* = 15) in all experiments performed. After surgery, all animals received penicillin G potassium (20,000 units) intramuscularly, to prevent infection.

### Behavioral Testing and Division of Rats into Groups

**Figure 1:**
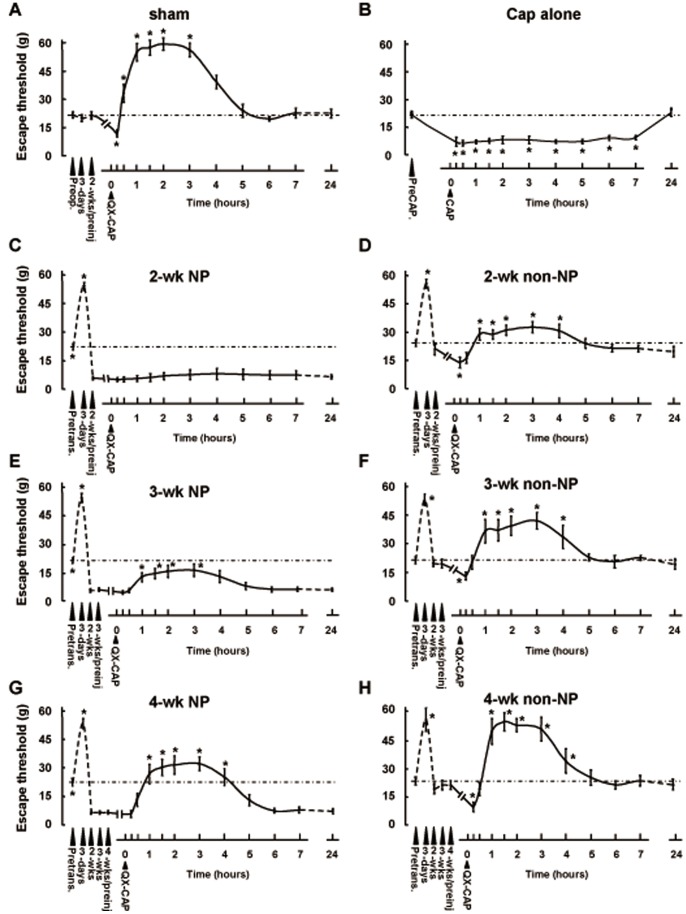
The effect of QX-CAP application on the escape threshold of NP and non-NP group at different time points after IAN transection. The changes in escape threshold following subcutaneous application of QX-CAP in sham-operated group (A); Only CAP injected sham-operated group (B); 2-weeks NP group (C); 2-weeks non-NP group (D); 3-weeks NP group (E); 3-weeks non-NP groups (F); 4-weeks NP group (G); 4-weeks non-NP group (H). The measurement were performed before the transection, 3 days after transection, 2, 3, and 4 weeks after transection/sham operation (depending on groups) and at various time points after injection of QX-CAP or CAP (n = 15 for each group, ANOVA followed by Dunnett’s test, *p<0.05). QX: QX-314; CAP: Capsaicin; Preop.: Preoperation; Preinj.: Preinjection; Pretrans.: Pretransection.

In daily sessions, rats were trained to stay in a plastic cage and keep their snout protruding through a hole in the cage wall during mechanical stimulation of the mental skin, using von Frey filaments (Touch-Test Sensory Evaluators; North Coast Medical, Inc., CA, USA). Touching and rubbing the rat’s mental skin without painful stimuli, by the shaft of von Frey filaments every day for 5–7 days (when the rats kept their snout protruded), allowed training the rats to keep their snout protruding through the hole for a long period of time. The force used, which brought upon escape behavior was determined and defined as the escape threshold (Fig 1). After successful training, the escape threshold was determined for the mental skin area, before and after IAN transection. The rats were free to escape following von Frey stimulus. Such escape behavior was defined as nocifensive. To determine the escape threshold, von Frey mechanical stimuli were applied to the mental skin in ascending and descending series of trials. The von Frey stimulus was applied 5 times in each series of trials. Escape threshold intensity was determined when the rats moved their heads away from the hole in at least one of the 5 stimuli. The average threshold intensity was calculated from the values after 2 ascending and 1 descending series of trials. Mechanical escape thresholds were measured at pre and 3 days, 2, 3, and 4 weeks post IAN transection.

The IAN-transected rats (IANx) were divided into neuropathic pain (NP) and non-neuropathic pain (non-NP) groups according to the following criteria: the rats that showed a mechanical escape threshold of ≤8 gram (g) after IAN transection were considered to have developed NP [20], [21], [22]. Each group was further divided into 3 subgroups, according to the time (in weeks) elapsed after IAN transaction and the escape threshold before QX-CAP administration. QX-CAP administration was performed at 2, 3 and 4 weeks following IAN transection. The rats that showed an escape threshold of ≤8 g at 2 weeks after IAN transection were defined as the 2-week NP group (*n* = 15). The rats that showed an escape threshold of ≤8 g at 2 weeks after IAN transection, which remained constant also at 3 weeks, were named the 3-week NP group (*n* = 15). The rats that showed an escape threshold of ≤8 g at 2 weeks after IAN transection, which remained constant also at 3 and 4 weeks, were defined as the 4-week NP group (*n* = 15). In the non-NP group (escape threshold following IAN >8 g), the rats were similarly divided into the 2-week non-NP (*n* = 15), 3-week non-NP (*n* = 15), and 4-week non-NP (*n* = 15) groups respectively, in order to evaluate behavioral responses in an extended period of between 2–4 weeks following transection similar to the NP groups.

**Figure 2:**
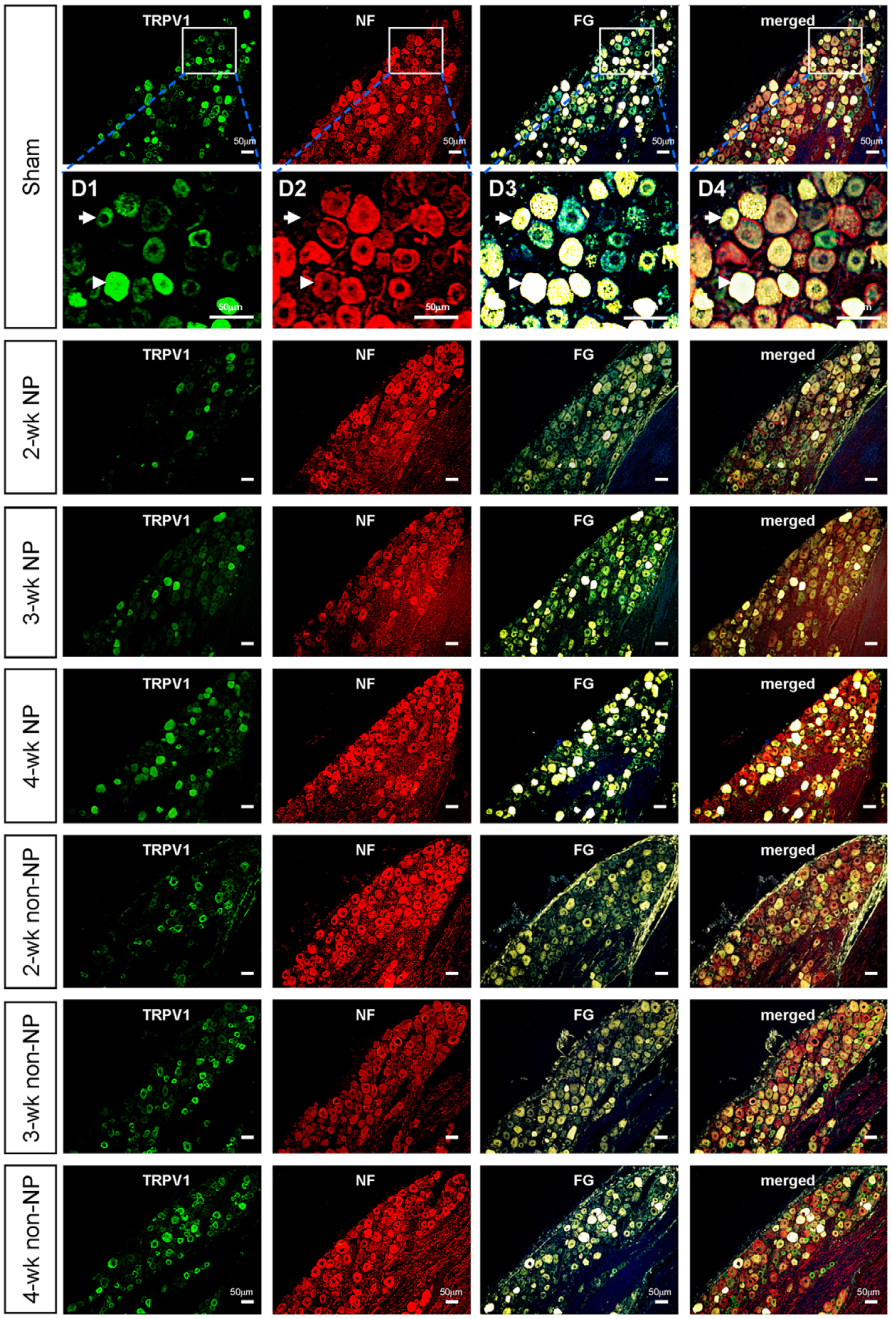
Photomicrographs of immunohistochemistry of TG cells labeled for TRPV1, NF200 and FG in sham-operated group and in 2-; 3-and 4-week NP groups and in 2-; 3- and 4 weeks non-NP groups. Expanded view of TG in the sham-operated group (D1–D4). Arrow points on an example of TRPV1^+^+FG^+^+NF^-^ cell. Arrowhead points on an example of TRPV1^+^+FG^+^+NF^+^ cell. Note that TRPV1-positive cells increased with time after transection. Scale bar: 50 µm.

### Drugs and Chemicals

N-(2,6-dimethylphenylcarbamoylmethyl) triethylammonium bromide (QX-314) (Sigma-Aldrich, St. Louis, USA) was used as a 2% solution in normal saline (0.9% NaCl in distilled water). Capsaicin solution (Wako Pure Chemical Industries, Ltd., Osaka, Japan) was prepared with Tween 20 (10%), ethanol (10%), and normal saline (80%). QX-314 was freshly prepared on the day of the experiment. Capsaicin solution was prepared every 15 days and kept in the refrigerator (4°C). Before commencement of individual experiments, capsaicin solution was equilibrated at room temperature for 30 min.

**Figure 3:**
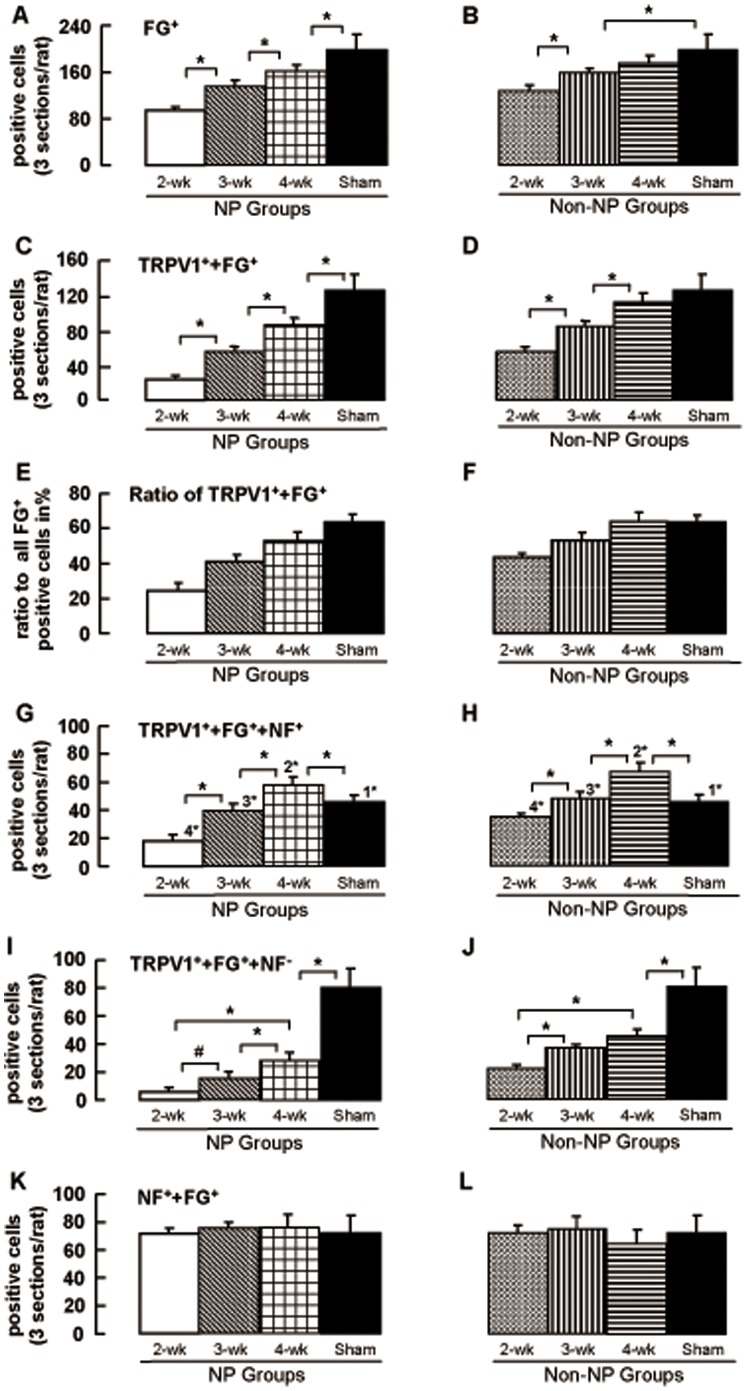
IAN transection both in NP and non-NP groups changes the expression profile of TRPV1 to myelinated neurons of a larger diameter. The total number of TG cells labeled for the fluoro-gold (FG^+^) (A: NP group, B: Non-NP group); TG cells that labeled for TRPV1 and FG (TRPV1^+^+FG^+^) in 2-; 3-and 4-week NP groups and in sham-operated group (C: NP group, D: Non-NP group). The ratio of TRPV1^+^+FG^+^ to all FG+ positive cells (E: NP group, F: Non-NP group). n = 5 for each group, (ANOVA followed by the Student–Newman–Keuls test, *p<0.05). The number of cells positive for TRPV1, FG and NF200 (TRPV1^+^+FG^+^+NF^+^) (G: NP group, H: Non-NP group); positive for TRPV1 and FG but not for NF200 (TRPV1^+^+FG^+^+NF^-^) (I: NP group, J: Non-NP group); positive for NF200 and FG (NF^+^+FG^+^) (K: NP group, L: Non-NP group) in 2-; 3-and 4-week NP groups and in sham-operated group revealed by immunohistochemistry. ANOVA followed by the Student–Newman–Keuls test. # indicates non-significant difference. TRPV1^+^+FG^+^+NF^-^ and TRPV1^+^+FG^+^+NF^+^ positive cells between the same groups are compared by paired t-test and the statistical significances are shown in the figure (G and H). 1*−4* indicate significant difference. 1: Sham TRPV1^+^+FG^+^+NF^+^ Vs TRPV1^+^+FG^+^+NF^-^, 2: 2-wk non-NP TRPV1^+^+FG^+^+NF^+^ Vs TRPV1^+^+FG^+^+NF^-^, 3: 3-wk non-NP TRPV1^+^+FG^+^+NF^+^ Vs TRPV1^+^+FG^+^+NF^-^, 4: 4-wk non-NP TRPV1^+^+FG^+^+NF^+^ Vs TRPV1^+^+FG^+^+NF^-^. p<0.05. n = 5 for each group.

### Injection of Drugs and Behavioral Testing

During each experimental session, preinjection mechanical escape thresholds of the mental skin area were measured ipsilateral to IAN transection. QX-314 (2%, 50 µl) with capsaicin (1 µg/µl, 30 µl) solution was subcutaneously injected into the mental skin area on the side ipsilateral to IAN transection, using a Hamilton microsyringe. In the sham-operated group, the solution of QX-314 with capsaicin or capsaicin was injected into the left mental skin area. Following injection, the escape threshold from the ipsilateral side was measured at 15 and 30 min, 1, 2, 3, 4, 5, 6, 7, and 24 h after injection. In the sham-operated group, the escape threshold from the left mental skin area was measured.

**Figure 4:**
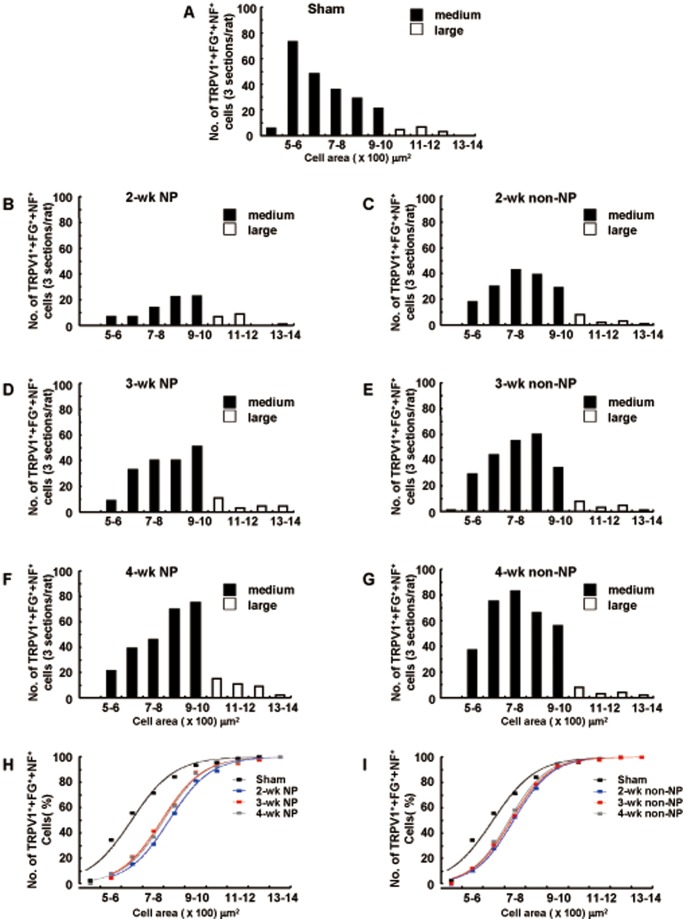
The pattern of distribution of TRPV1 was altered in non-NP groups. The distribution area of TRPV1^+^+FG^+^+NF^+^ positive cells for all experimental groups. A cell area >1000 µm^2^ was considered large, while that <1000 µm^2^ was considered medium. Note that most of the cells were in the medium range, and the peak distribution shifted to the right in the transected groups. n = 5 for each group.

**Table 1:** The distribution analysis of cell area of TRPV1^+^+FG^+^+NF^+^ in different groups.

Sigmoidal fit:
Sham 2-wkNP 3-wk NP 4-wk NP 2-wk non-NP 3-wk non-NP 4-wk non-NP
x(0) (Mean±SD) 644.7±13 822±4.6 788.2±6.6 796.7±5.5 742.4±3.8 731.4±4.1 721.6±5.3
d(X) (Mean±SD) 101.6±11.7 97.9±4 96.9±6 95.9±4.9 90.2±3.4 90.6±3.4 88.1±4.7

x(0): Sham vs. 2 weeks NP, p<0.001; Sham vs. 3 weeks NP, p<0.01; Sham vs. 4 weeks NP, p<0.001; Sham vs. 2 weeks non NP, p<0.01; Sham vs. 3 weeks non NP, p<0.05; Sham vs. 4 weeks non NP, p<0.05. There was no significant difference in d(x). One-way ANOVA with post-hoc Bonfferoni, n = 5 for all groups.

### Immunohistochemistry

Five rats were chosen randomly from each group and used for the immunohistochemical experiments. Fluoro-Gold (FG) (2%, 10 µl) was subcutaneously injected into the mental skin area under sodium pentobarbital (50 mg/kg, administered intraperitioneally) anesthesia, 2 days before perfusion and used for retrograde labeling of neurons in order to give an estimate of the extent of regeneration following the IAN procedure. Then the rats were deeply anesthetized with sodium pentobarbital and perfused with 200 ml of normal saline followed by 500 ml of 4% paraformaldehyde. The trigeminal ganglion (TG) was removed and post-fixed in 4% paraformaldehyde, for 2 days and the tissue was then transferred to a solution of 20% sucrose in phosphate-buffered saline (PBS) for several days for cryoprotection. Sections (16 µm in thickness) were cut using a cryostat, and every fifth section was mounted on MAS-coated glass slides (Matsunami Glass Ind., Ltd., Osaka, Japan). After washing with PBS, the sections were incubated at room temperature with 3% normal goat serum (NGS) in 0.01 M PBS with 0.3% Triton X-100, for 1.5 hours. They were then coincubated overnight at 4°C with a combination of rabbit anti-TRPV1 antibody (1∶200; Alomone Labs Ltd., Israel), which was diluted with 3% NGS in 0.01 M PBS with 0.3% Triton X-100, and mouse monoclonal anti-neurofilament 200 (NF200) antibody (1∶1000; Sigma-Aldrich), which was diluted with 3% NGS in 0.01 M PBS with 0.3% Triton X-100. The sections were washed 3 times with PBS and then incubated with goat anti-rabbit IgG (Alexa Fluor 488, 1∶1000; Invitrogen, USA) and goat anti-mouse IgG (Alexa Fluor 568, 1∶1000; Invitrogen) for 2 h at room temperature. After washing with PBS, the slides were coverslipped with Vectashield mounting medium (Vector Laboratories, Inc., USA). The stained slides were viewed and imaged using a camera attached to a Biozero BZ-8000 fluorescent microscope (Keyence Corp., Japan). The area viewed at 100x (700×850 µm) at the root of the third branch of TG was used for counting labeled cells. For each rat, three sections (one with the largest number of labeled cells and the next two sections) were selected for counting.

The cell area was measured using ImageJ software (NIH Image, USA) for cells expressing TRPV1, NF200, and FG. A cell area >1000 µm^2^ was considered large, while that <1000 µm^2^ was considered medium and <400 µm^2^ was considered small [20].

### Statistical Analysis

For the analysis of the last significant time point of the behavioral effect of QX-CAP, one way analysis of variance (ANOVA) followed by Dunnett’s test were used. For comparison of the magnitude of the effect, the area under curve (AUC) was calculated and compared using a t-test. In addition, the comparison between different groups was tested statistically using two-way ANOVA. Immunohistochemical data were analyzed using one-way ANOVA followed by the Student–Newman–Keuls test. To compare TRPV1 expressing regenerated myelinated and non-myelinated neurons between the same groups paired t-test was used. To compare changes in distribution of the area of cells expressing TRPV1 channels, 3 slices from each rat for each group were compared. Since the distribution of cell areas did not follow Gaussian distribution (analyzed by Shapiro-Wilk and Kolmogorov Smirnov test), the cumulative probability of the areas of the examined cells was calculated. Data were then fit by a Boltzmann relationship: y = (A_1_– A_2_/(1+ exp [(x-x(0))/dx] = A_2_), and the x(0) (which is the cell area at which of 50% of examined cells express TRPV1 channels), was calculated for each group and compared using one-way ANOVA with post-hoc Bonferroni. *p* value <0.05 was considered as statistically significant. Data are expressed as mean ± standard deviation.

## Results

Application of capsaicin, by virtue of its activation of TRPV1 channels, facilitates the entry of the permanently charged membrane impermeant sodium channel blocker, QX-314, selectively into nociceptive neurons, and thereby produces pain-selective analgesia [15], [16], [17]. Here we examined whether this platform could be also used to attenuate neuropathic pain resulting from nerve injury. We wanted to explore the dynamic effective range of the QX-CAP combination in sham-operated as well as those animals which underwent inferior alveolar nerve transection (IANx) and developed neuropathic pain (NP). To this end, we measured the mechanical escape threshold of the mental region of rats before and after subcutaneous application of QX-CAP into this area, at different time points after IANx. In sham operated animals, subcutaneous application of QX-CAP lead to significant and robust increase of the escape threshold, which lasted for 3.5 hours (post-hoc Dunnett’s test) and then decreased to near basal levels as before sham operation (Fig 1A), though only CAP application in sham rats showed that the escape threshold reduction lasted over 6 hours (Fig 1B). This was not the case with the NP groups tested before and after QX-CAP application. In the 2-week NP group (the rats that showed a mechanical escape threshold of ≤8 g 2 weeks after IANx) there was a transient increase in threshold which is attributed to deinneravation followed by a steady state decrease in the threshold prior to application. Following QX-CAP application, there was no significant increase in response to mechanical stimuli after the initial decrease in threshold (p>0.05, one-way ANOVA followed by Dunnett’s test) (Fig 1C). In the 3-and 4-week NP groups, the profile of change in mechanical threshold before QX-CAP application was similar to that of the 2 week group (Fig 1E, G). However, application of capsaicin and QX-314 at 3 and 4 weeks transiently reversed the IANx-mediated decrease in escape threshold (Fig 1E, G). In the 3-week NP group, the QX-CAP mediated increase in the escape threshold lasted for 3 hours and was significantly higher than that of the 2-week NP group (AUC_3-weeks NP_ = 2619.6±811; AUC_2 weeks NP_ = 1495.5±1074, p = 0.017, t-test, p<0.001, two way ANOVA) (Fig 1C, E) but significantly lower than the effect shown for the sham operated group (AUC_Sham_ = 9317±1069, p<0.001, t-test; p<0.001, two way ANOVA) (Fig 1A, E). In the 4-week NP group, the observed effect lasted also for 3 hours (post-hoc Dunnett’s test) and was significantly higher than the 3-week NP group (p<0.001, AUC analysis; p<0.001, two way ANOVA) (Fig 1E, G) but still significantly lower than that of the sham operated group (p<0.001 AUC analysis, p<0.001, two way ANOVA). To further explore the reasons for these results, we looked into the extent of regeneration, as evidenced by retrograde fluoro-gold FG^+^ labeling. The lack of response in the 2 week group could not be explained by lack of the fibers innervating the mental skin, since we observed significant regeneration (Fig 2 and Fig 3A). We found that the extent of regeneration increased as time went by from the 2 week group and up to the 4 week group, albeit a smaller extent than the sham operated group.

Since the effect of QX-CAP strongly depends on activation of TRPV1 channels, we further explored whether the lack of response in the 2 week group could be due to the diminished TRPV1 expression in this NP group. We found that the relative amount of TRPV1 expressing cells among FG^+^ trigeminal ganglion neurons in the 2-week NP group was, however, significantly smaller than in the sham operated group (the ratio of TRPV1^+^+FG^+^ to all FG ^+^ positive cells in the 2 week NP group was 24.5±4.4% and the ratio of TRPV1^+^+FG^+^ to all FG ^+^ positive cells in the sham operated group was 63.5±3.9%) (Fig 2 and Fig 3C, E). Moreover, the ratio of TRPV1^+^+FG^+^ to all FG^+^ positive cells in the 3-week NP group was higher than the 2-week NP group (41±3.8%) (Fig 2 and Fig 3E). In the 4-week NP group, the ratio was even higher than in the 3-week NP group (52.8±5.2%) (Fig 2 and Fig 3E), but still did not reach the level of the sham operated group (63.5±3.9%). We found a clear positive correlation between the extent of the QX-CAP effect in the different groups and increased TRPV1 expression in the regenerated neurons (Fig 2 and Fig 3C, E). Since there was no effect for QX-CAP at 2 weeks after IANx, we assumed that QX-314 alone cannot explain the results shown in the other groups. It is also apparent that at 2 weeks there were enough sensory fibers and sufficient excitability to allow for the decreased painful threshold. Thus, we conclude that the effect of QX-CAP is the result of increased TRPV1 expression in the regenerating neurons and that the effect is targeted blockade of sodium channels via TRPV1 channels as demonstrated in other pain related models [15], [16], [17], [18]. This approach can be used as an effective pain treatment taking into account the time constrains of TRPV1 expression along the timeline of regeneration after nerve injury. This result also allows for indirect measurement of TRPV1 expression as evident from changes in pain related behavior (i.e. threshold).

In addition to realizing the effect of TRPV1 expression on the behavior, we explored its dynamic expression profile between different neuronal cell types. To this end we used myelinated neuron marker NF-200 (NF) to examine the effect of IANx on the expression of TRPV1 channels among myelinated (TRPV1^+^+FG^+^+NF^+^) and non-myelinated (TRPV1^+^+FG^+^+NF^-^) regenerated neurons. As expected from previous results, the number of TRPV1^+^+FG^+^+NF^-^ cells, which can account for the pain sensing C-fibers was very low in the 2-week NP group (5.9±3.5) compared to the sham (80.4±13.5) (Fig 2 and Fig 3I). Surprisingly, although the total number of TRPV1^+^+FG^+^ cells in the 3-week NP group was significantly higher than in the 2-week NP group (Fig 2 and Fig 3C), the number of TRPV1^+^+FG^+^+NF^-^ neurons wasn’t statistically different from the 2-week NP group (5.9±3.5 vs. 16.1±5.5, respectively, p>0.05, one-way ANOVA followed by the Student–Newman–Keuls test) (Fig 2 and Fig 3I). However we found that most of the TRPV1 expression was attributed to myelinated neurons (Fig 2 and Fig 3I). Indeed, while in the sham-operated group the number of TRPV1^+^+FG^+^+NF^-^ cells (63.8% of the total TRPV1^+^+FG^+^ cells (Fig 2 and Fig 3C, I) was significantly higher than that of TRPV1^+^+FG^+^+NF^+^ cells (36.2% of the total TRPV1^+^+FG^+^ cells (Fig 2 and Fig 3C, G), in the NP groups, the number of TRPV1^+^+FG^+^+NF^-^ cells was significantly lower than that of TRPV1^+^+FG^+^+NF^+^ cells (67%–76% across the different groups vs. 23%–32% across the different groups, respectively). The number of TRPV1^+^+FG^+^+NF^-^ gradually increases (16.1±5.5 vs. 28±5.9, for 3-weeks NP and 4-weeks NP, respectively, p<0.05, one-way ANOVA followed by the Student–Newman–Keuls test) (Fig 2 and Fig 3I). Even the 4 week NP group had significantly lower TRPV1 expression than in the sham operated group (28±5.9 vs. 80.4±13.5 in 4-week NP and sham operated groups, respectively, p<0.001, one-way ANOVA followed by the Student–Newman–Keuls test) for non myelinated neurons (Fig 2 and Fig 3I).

The number of the TRPV1^+^+FG^+^+NF^+^ cells also increased after NP injury to levels higher than the sham-operated group (Fig 2 and Fig 3G) (57.8±5.9 vs. 45.6±5.1, 4 week group and sham operated group respectively, p<0.01, one-way ANOVA followed by the Student–Newman–Keuls test) but the amount of myelinated regenerated neurons (NF^+^+FG^+^) without TRPV1 did not change with time (Fig 2 and Fig 3K).

Collectively, these data show that the pattern of expression of TRPV1 channels changes after nerve injury, such that TRPV1 channel expression shifts to become more prominent in myelinated neurons, the importance of which will be discussed down below.

As stated in the above method section, the animals were divided into NP and non-NP groups according to which animals developed nocifensive behavior (i.e. change in threshold occurring after IAN). It was important to explore why certain animals did not show a decrease in the mechanical threshold and whether this observation has any link to TRPV1 expression profiles following IANx.

Similarly to the NP groups, the number of TRPV1^+^+FG^+^ cells gradually increased with time after the transection (Fig 2 and Fig 3C). However the level of TRPV1-expressing regenerated cells was significantly higher in non-NP groups than in NP groups (p<0.001 for all NP vs. non NP groups, one-way ANOVA followed by the Student–Newman–Keuls test). In the 2 week non-NP group about 40% (43.6±2.2%) (Fig 2 and Fig 3F) of the regenerated cells expressed TRPV1, whereas in the 2 week NP group, only about 25% of the regenerated trigeminal neurons expressed TRPV1 (24.5±4.4%). The number of TRPV1^+^+FG^+^ neurons in the 3-week non-NP group was higher than that of the 2-week non-NP group (84.4±6.2 vs. 56±5.2, respectively, p<0.001, one-way ANOVA followed by the Student–Newman–Keuls test) but significantly lower than in the 4-week non-NP group (112.2±10.4) (p<0.001, one-way ANOVA followed by the Student–Newman–Keuls test), and reached near control levels (in sham operated group, 126±18.2) 4 weeks after injury (p>0.05, one-way ANOVA followed by the Student–Newman–Keuls test; Fig 2 and Fig 3D). Moreover the absolute number of regenerating neurons increased with time, reaching near sham levels at 4 weeks (Fig 2 and Fig 3D). However the amount of regenerating neurons at any time point was significantly higher when compared to levels seen for the NP groups, which might hint to a larger and more robust behavioral effect when applying QX-CAP in conjunction to TRPV1 levels per regenerating nerves (compare Fig 3F to Fig 3E). Based on these results and those of Fig 1, we speculated that this expression profile would lead to more profound effects of QX-CAP application in terms of increasing threshold levels. Indeed this was the case. Application of QX-CAP to the mental skin of non-NP animals produced a significant increase of escape threshold in all non-NP groups (Fig 1D, F, H), including the 2 week group, for which there was no effect in the NP animals (Fig 1C vs. Fig 1D). It is important to mention that the behavioral profile in general between all groups was the same. The initial increase in threshold preceding the IAN transection was also evident in the non-NP groups, indicating that the procedure was complete and the difference in effects seen between NP and non-NP groups could not be the result of a difference in the IAN procedure. However the question of difference in threshold between the groups prior to QX-CAP application still remains elusive. The duration of QX-CAP mediated effect was similar in all non-NP groups and also when compared to the NP groups, namely a transient 3 hour effect which then returned back to baseline (p<0.05, one-way ANOVA followed by Dunnett’s test). The magnitude of the blockade in the 2-week non-NP group was not significantly different from that seen in the 3-week non-NP group, according to AUC (AUC_2-weeks non-NP_ = 5713±1327 vs. AUC_3-weeks non-NP_ = 6590.1±1696) (p>0.05, t-test) (Fig 1D, F). However, analysis performed by two-way ANOVA with post-hoc Bonferroni comparing specific time points, did show a statistical significant difference between the groups (30 min, 1, 1.5, 2 and 3 hour post injection). However, the effect seen in the 4-week non-NP group was significantly higher than in the 2 and 3 week non-NP groups (AUC_4-weeks non-NP_ = 8361.5±1637; p<0.001, t-test; p<0.001 two way ANOVA) (Fig 1D, F, H).The effect of QX-CAP mediated blockade in the 4-week non-NP group was similar to the effect on sham operated animals according to AUC analysis of data (AUC_Sham_ = 9317±1069; p>0.05, t-test) but different when using specific time point in two-way ANOVA analysis (Fig 1A, E). The effect in the sham operated group was higher than that of the 4-week non-NP group at 30 min, 2 and 4 hour post injection. In conclusion we see that the level of TRPV1 expression correlates to magnitude of QX-CAP application across all non-NP groups.

We examined whether the pattern of distribution of TRPV1 channels is altered in non-NP nerve injury model and how this compares to the NP groups. In general the pattern and change in profile is similar. The non-NP groups exhibit increasing levels of TRPV1 with time in myelinated regenerated neurons, surpassing the level as shown in the sham group (Fig 2 and Fig 3H). Thus, at 4-weeks, the number of TRPV1^+^+FG^+^+NF^+^ cells (67±6.5) was significantly higher than in the sham operated group (45.6±5.1, p<0.001, one-way ANOVA followed by the Student–Newman–Keuls test) (Fig 2 and Fig 3J). The level of TRPV1 in non myelinated neurons also increased with time but did not reach sham levels (Fig 2 and Fig 3J). The number of TRPV1^+^+FG^+^+NF^-^ neurons at 4 weeks (45.2±4.6) was approximately half of the sham operated group (80.4±13.5) (Fig 2 and Fig 3J) (p<0.001, one-way ANOVA followed by the Student–Newman–Keuls test). Similar to NP groups, the proportion of the TRPV1^+^+FG^+^+NF^+^ cells was significantly higher than that of TRPV1^+^+FG^+^+NF^-^ cells (56%–61% across the different groups (Fig 3D, H) vs. 38%–42% across the different groups (Fig 3D, J, respectively).

Just as observed for the NP groups, here too, the number of myelinated regenerated neurons without TRPV1 is high (comparable to sham) and does not change with time (Fig 2 and Fig 3L). These data indicate that the sensitivity or absence of sensitivity which different groups (NP and non NP groups) show to painful stimuli and development of neuropathic pain behavior, are not necessarily linked to the biogenesis like process which ultimately shapes the TRPV1 expression profile following IANx.

Since myelinated neurons were the major cell type to express TRPV1, we explored the distribution of expression of TRPV1 as a function of cell area (Fig 4). This would represent cell size and give a more accurate indication of sub cell type. In all experimental groups, most of the TRPV1^+^+FG^+^+NF^+^ cells were found to be medium sized with averaged cell body areas of less than 1000 µm^2^ (Fig 4). However, the peak cell area distribution shifted to the right following both NP (Fig 4B,D,F,H) and non-NP (Fig 4C,E,G, I) IANx. These data suggest that nerve injury with or without altered pain sensation changes the expression profile of TRPV1 channels, not only to myelinated neurons but of a larger diameter (table1).

## Discussion

In the current study, we measured, for the first time, the functional dynamic expression of TRPV1 during the regeneration process of transected IAN nerve in rats. Using the facilitated entry of QX-314 through the TRPV1 channel activated by capsaicin, we demonstrated that increasing amounts of TRPV1 expression would allow for functional and selective blockade of painful sensation following neuropathic based nerve injury (i.e. IAN). For the first time, we could interpret the amount of TRPV1 expression as an indication of successful anesthetic effect for QX-CAP and vise versa. We are now able in this model to predict the effectiveness of such a strategy at different time points following nerve injury. We can now also use the behavioral tests as a bio assay to predict relative TRPV1 expression levels.

Peripheral nerve transection followed by close apposition of the cut ends leads to axon regeneration and subsequent re-innervations of the target tissue. Depending on the regeneration distance, this process can take several weeks to months [23], [24], [25], [26] and involves various molecular and biophysical changes in sensory neurons [27], [28], [29], [30], [31], [32], [33], [34], [35]. For example, nerve injury is known to induce alteration in receptors, ion channels, neuropeptides, signal transduction molecules, and growth related proteins, as well as to increase the spontaneous activity and receptive field of sensory neurons [22], [36], [37], [38], [39], [40], [41], [42], [43], [44], [45], [46], [47], [48]. These changes could be attributed to the injury itself and/or the altered environment encountered by regenerating axons at the injury site and/or the target tissue, and lead to a neuropathic condition characterized by allodynia or hyperalgesia [22], [28], [39], [49]. The effectiveness of QX-CAP injection may differ under these abnormal conditions. In our study, we evaluated the effectiveness of this combination in the sham-operated group at 2 weeks after operation, which can be considered as the control condition, and in various IAN-transected groups. In the IAN-transected groups, we evaluated the effectiveness under NP (indicated by a decrease in mechanical escape thresholds) and non-NP conditions (where the mechanical escape threshold did not decrease). We also evaluated the effectiveness of QX-CAP at various time points after transection as the underlying environment may change with time and such a study has not yet been performed. Evaluating the effect of QX-CAP injection in various conditions allowed us to comprehend the functional expression of TRPV1 in those conditions and to evaluate the outcome of such manipulations.

In agreement with this view, QX-CAP injection showed variable local anesthetic effects under different conditions. In the sham-operated group, QX-CAP injection caused a significant increase in the mechanical escape threshold for 3.5 h (Fig 1A). The escape threshold reduction lasted over 6 hours when only CAP was injected the sham-operated group. This result implies that capsaicin-induced sensitization does not evoke in the capsaicin concentration we used.

The escape threshold increased to more than 2 times the preinjection level and in many cases, the mental skin was insensitive to even the 60 g von Frey stimulus. Under non-NP conditions at 4 weeks after IAN transection, the threshold was found to be similar to that of the sham-operated group (Fig 1H). These findings indicate that QX-CAP injection was highly effective in these 2 groups. Under NP conditions, at 3 and 4 weeks after transection, a significant increase was observed in the escape thresholds, indicating that QX-CAP produced an analgesic effect in these groups, similar to the non-NP groups. We observed that the effectiveness of QX-CAP injection depends on time elapsed from transection and are positively correlated to increasing expression levels of TRPV1. These observations are true for both NP and non-NP groups. These results are in line with previous studies performed on naive animals, whereby QX-CAP injection was shown to produce an effective, nociceptor-specific local anesthesia effect? [15], [16], [17]. QX-CAP injection into rat hind paws resulted in a long-lasting increase in mechanical and thermal nociceptive thresholds [16]. In a recent study, the co-application of these drugs was observed to be effective in blocking pain signals in the rat trigeminal system [17]. These studies showed that QX-314 entered through the activated TRPV1 channel.

In the current study we also evaluated the type of regenerated neurons in which TRPV1 is expressed after nerve transection. As mentioned above, we used NF200 as a marker for myelinated neurons (including Aβ and Aδ) and also injected FG (a retrograde tracer) into the mental skin area to identify the regenerated neurons. We found that the regenerated neurons expressing TRPV1 gradually increased over time after IAN transection (Fig 3C, E and 3D, F). However, in rats that developed NP, the number of regenerated neurons expressing TRPV1 was smaller compared to those with non-NP at the same time points. In the 4-week non-NP group, the number of regenerated neurons expressing TRPV1 was similar to that in the sham-operated group. Comparison of these immunohistochemical findings with behavioral data suggested that the variable anesthetic effect of QX-CAP injection appears to be due to the availability of TRPV1 receptors on regenerated neurons. In rats with NP at 2 weeks after transection, the number of regenerated neurons expressing TRPV1 was smaller compared to sham and the corresponding non-NP group of 2 weeks, and the corresponding behavioral study showed that QX-CAP injection was not effective. We hypothesize that entry of QX-314 was limited due to reduced TRPV1 expression and therefore not sufficient to make the combination effective in the behavioral readout test. In the 3-and 4-week NP groups, TRPV1 was shown to be expressed in higher numbers in myelinated (medium-sized) neurons and the analgesic effect that we observed in the 3-and 4-week NP groups might have been mediated by entry of QX-314 via TRPV1 channels in these neurons, as well as through non-myelinated neurons with TRPV1. The smaller effect of QX-CAP injection in the non-NP group at 2 and 3 weeks after transection is also probably due to the reduced availability of TRPV1 in regenerated neurons. An interesting question arises as to why certain animals develop decreased threshold (the NP groups) and other do not (the non-NP groups) although they both underwent the same procedure and to the same extent? The results show that TRPV1 levels are generally higher in the non-NP groups even at 2 weeks post IANx. Many studies have associated painful sensation to TRPV1 expression as this channel is considered to be the transducer of painful stimuli. Therefore one would expect that higher levels of TRPV1 would convey higher sensitivity to painful stimuli. Could this difference in profile also indicate whether or not an animal will develop neuropathy based painful stimuli? Could it be related also to the extent of regeneration between the groups which is also higher in the non-NP groups? These points need further investigation. Insight into this subject has great relevance when crossing over to the clinic and will be of great importance in developing new strategies which distinguish between different groups but also offer the right protocol to diminish pain for those which unfortunately fall into the NP groups.

The immunohistochemical study revealed that C-fibers (non myelinated TRPV1 expressing neurons) were slow to regenerate after transection, and this regeneration was even slower under NP conditions compared to non-NP conditions. This data is in conjunction with previous reports showing that the C-fibers of injured nerves take longer to regenerate than myelinated A-fibers [23], [25], [50], [51]. Saito and colleagues showed that regenerated TG neurons with small diameter were significantly reduced at 14 and 60 days after IAN transection [21], though it has been reported that TRPV1 function is upregulated in IB4-positive sensory neurons (small neurons) [52]. They demonstrated that IAN-transected rats showed a profound reduction to thermal stimuli. Thermal sensory information is predominantly conveyed by C-fibers [53], [54]. Therefore, reduced sensitivity to thermal stimuli indicates a reduced number of C-fibers after transection (see also [20]). Our study also showed that TRPV1 expression shifted to myelinated fibers after transection. In the sham-operated group, TRPV1 was mostly expressed in small size C-fibers. However, in the IAN-transected groups, TRPV1 was mostly expressed in medium sized-neurons, in both NP and non NP groups. These results fit well with previous studies which also showed TRPV1 expression shifting to myelinated neurons in the dorsal root ganglion of rats with chronic inflammatory conditions induced by Freund’s complete adjuvant [55], [56]. Similar shifting has also been reported in animal models for diabetic neuropathy and bone cancer pain [57], [58], [59]. Although medium sized myelinated fibers are traditionally not involved in pain detection, these fibers have been reported to be involved in NP after nerve injury [20], [21], [39], [48], [50], [60]. Background activity, mechanically evoked responses and discharge of Aδ-fibers increased significantly in IAN-transected rats compared with naïve rats [20]. In other studies, it has been reported that after nerve injury, A-fibers were able to produce substance P and calcitonin gene-related peptide (CGRP), usually released from C-fibers [61], [62], [63]. Central sprouting of myelinated fibers has also been postulated to be an underlying cause of NP after nerve injury [48], [60], [64], [65]. Therefore, it is certainly plausible that entry of QX-314 into myelinated fibers via TRPV1 activated by capsaicin, may block abnormal activity of those fibers under neuropathic conditions and that these fibers have a role in NP related behavior. A detailed knowledge of expression profiles together with profound understanding of myelinated and non-myelinated neurons contributing to neuropathy, will lead to successful development of strategies in attenuating neuropathic pain.
